# Change in Sucrose Cleavage Pattern and Rapid Starch Accumulation Govern Lily Shoot-to-Bulblet Transition *in vitro*

**DOI:** 10.3389/fpls.2020.564713

**Published:** 2021-01-14

**Authors:** Yun Wu, Ziming Ren, Cong Gao, Minyi Sun, Shiqi Li, Ruihan Min, Jian Wu, Danqing Li, Xiuyun Wang, Yanping Wei, Yiping Xia

**Affiliations:** ^1^Genomics and Genetic Engineering Laboratory of Ornamental Plants, Department of Horticulture, College of Agriculture and Biotechnology, Zhejiang University, Hangzhou, China; ^2^Department of Landscape Architecture, School of Civil Engineering and Architecture, Zhejiang Sci-Tech University, Hangzhou, China; ^3^Beijing Key Laboratory of Development and Quality Control of Ornamental Crops, Department of Ornamental Horticulture and Landscape Architecture, China Agricultural University, Beijing, China; ^4^Agricultural Genomics Institute at Shenzhen, Chinese Academy of Agricultural Sciences, Shenzhen, China

**Keywords:** lily, bulblet formation, starch synthesis, cell wall invertase, sucrose synthase, cytological observation, symplastic unloading, 5(6)-carboxyfluorescein diacetate

## Abstract

In bulb crops, bulbing is a key progress in micropropagation and is the feature that most distinguishes bulbous crops from other plants. Generally, bulbing involves a shoot-to-bulblet transition; however, the underlying mechanism remains elusive. We explored this process by tracking the shoot-to-bulblet transition under different culture conditions. Rapid starch accumulation occurred at 15 days after transplanting (DAT) in the bulblet-inducing treatments as confirmed via histological observations and the significant elevation of starch synthesis related-gene transcription, including *LohAGPS*, *LohAGPL*, *LohGBSS*, *LohSS*, and *LohSBE*. However, for shoots that did not transition to bulblets and maintained the shoot status, much higher soluble sugars were detected. Interestingly, we observed a clear shift from invertase-catalyzed to sucrose synthase-catalyzed sucrose cleavage pattern based on the differential expression of *LohCWIN* and *LohSuSy* during the key transition stage (prior to and after bulbing at 0–15 DAT). Shoots that transitioned into bulblets showed significantly higher *LohSuSy* expression, especially *LohSuSy4* expression, than shoots that did not transition. A symplastic phloem unloading pathway at the bulblet emergence stage (15 DAT) was verified via the 6(5)-carboxyfluorescein diacetate fluorescent tracer. We propose that starch is the fundamental compound in the shoot-to-bulblet transition and that starch synthesis is likely triggered by the switch from apoplastic to symplastic sucrose unloading, which may be related to sucrose depletion. Furthermore, this study is the first to provide a complete inventory of the genes involved in starch metabolism based on our transcriptome data. Two of these genes, *LohAGPS1.2b* and *LohSSIIId*, were verified by rapid amplification of cDNA ends cloning, and these data will provide additional support for *Lilium* research since whole genome is currently lacking.

## Introduction

*Lilium* is a monocotyledonous genus belonging to the Liliaceae family and has high ornamental and economic value. Lily bulbs are imbricate (scaly) bulbs, which is a type of true bulb in which the succulent leaf scales are not covered by a tunic. ‘Sorbonne’ (short for Loh) is an Oriental hybrid that is one of the four horticulturally important hybrid groups, and it is the most popular lily cultivar worldwide, especially in China (Kamenetsky and Okubo, 2012). It features large vibrant pink flowers with white margins and dark pink speckles. Recently, extensive Illumina sequencing-based technologies have been applied to investigate many important biological processes of this Oriental cultivar, such as floral scent production (Du et al., 2017a), vernalization (Liu et al., 2014; Li et al., 2016; Gu et al., 2020), and flower color formation (Zhang et al., 2015), thus confirming the plant’s importance.

Micropropagation has the potential to produce large numbers of high-quality plantlets in a short period of time and is already being commercially applied in lily production. Producing lily bulblet in tissue culture is advantageous because the bulblets are easy to handle and acclimate better than lily shoots after being transferred to the soil environment, which is the main difference between lily and other non-bulbous herbaceous and woody ornamental plants during tissue culture (de Klerk, 2012). The predominant *in vitro* propagation method for lily through direct organogenesis is via adventitious shoot formation, which normally uses excised scales as the primary propagules. The other method is via adventitious bulblet formation. Some flower bulbs, such as the tulip and iris, initially form only shoots *in vitro*. All these shoots need to undergo a bulbing process for later successful transfer to *ex vitro* conditions for the reasons stated above (de Klerk, 2012; Askari et al., 2018). For example, ‘Sorbonne’ usually develops cluster shoots first and forms bulblets afterward (Wu et al., 2017), which ensures a higher propagation coefficient than direct adventitious bulblet formation. In other words, the shoot-to-bulblet transition is an imperative key step for successful flower bulb micropropagation.

To date, many studies have focused on the optimization of culture conditions for lily shoot induction or bulblet formation (bulbing/bulbification) *in vitro* (Podwyszyńska, 2012). For instance, the explant type (Nhut, 1998), explant collection season (Robb, 1957), basal medium composition (Lian et al., 2002a), plant growth regulators (PGRs) (Kumar et al., 2007; Wu et al., 2017), sucrose concentration (Varshney et al., 2000; Azadi and Khosh-Khui, 2007; Gao et al., 2018), photoperiod (Lian et al., 2003), light quality (Lian et al., 2002b), temperature (Saadon and Zaccai, 2013), and mild abiotic stresses (Askari et al., 2016) have been studied. It is generally agreed that a high cytokinin/auxin ratio is beneficial to shoot initiation when bulb scales are used as explants (Jin et al., 2014; Sahoo et al., 2018), while sucrose is beneficial for bulbing (Podwyszyńska, 2012; Askari et al., 2018; Gao et al., 2018). At the physiological level, we previously reported that two different lily bulblet-promoting conditions *in vitro*, namely, low humic acid (HA) and paclobutrazol (PBZ) concentrations, were both strongly correlated with higher starch synthetic enzyme activities at various growth stages (Wu et al., 2016, 2019a). However, the comprehensive mechanism involved in *in vitro* bulblet formation remains largely unknown.

Generally, lily bulblet formation from shoots consists of two main biological events: the onset of the bulblet, which normally occurs during early developmental stages, and bulblet filling, which occurs in later stages. The lily scale consists of two kinds of reserve polysaccharides, glucomannan and starch; and the latter is the principal kind, accounting for approximately 85% of the scale dry matter (Matsuo and Mizuno, 1974; Ranwala and Miller, 2008). The bulblet filling process is characterized by the gradual accumulation of starch, which can be used as a nutrient reserve in later growth stages (Li et al., 2014). Unlike the filling step, no detailed attempts have been made to study the initial bulblet morphogenesis step, *viz.*, the shoot-to-bulblet transition, which is required for further scale filling step to occur. Similarly, the different characteristics of bulblet-inducing and bulblet-non-inducing bulblet (due to the problems in the shoot-to-bulblet transition process) conditions have not been compared. Conversely, in potato, which is a model storage organ species for studying tuberization and source-sink regulation, non-swelling stolons have commonly been used in different studies (Muñiz García et al., 2017; Abelenda et al., 2019).

**FIGURE 1:**
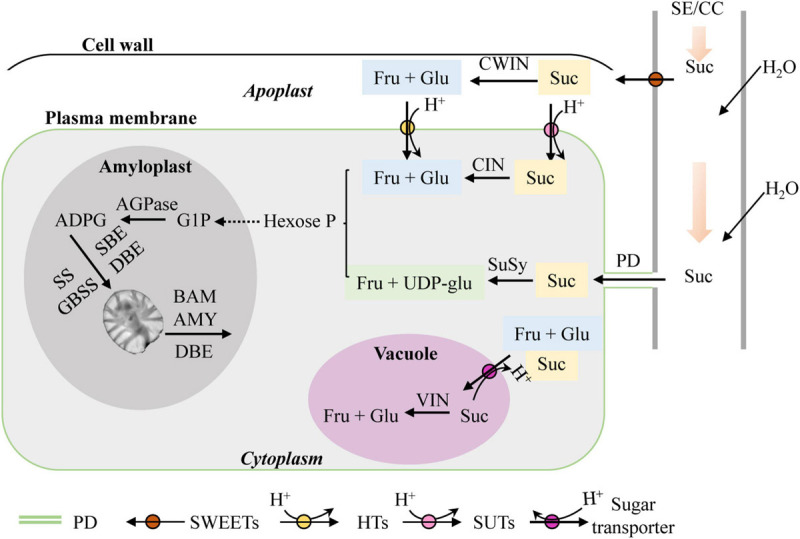
Sucrose and starch metabolism in sink tissues (adapted from Bahaji et al., 2014; Ruan, 2014). SE/CC, sieve element/companion cell; Suc, sucrose; CWIN, cell wall invertase; Fru, fructose; Glu, glucose; SWEET, sugars will eventually be exported transporter; PD, plasmodesma; SUT, sucrose transporter; HT, hexose transporter; CIN, cytoplasmic invertase; VIN, vacuolar invertase; SuSy, sucrose synthase; UDP-glu, uridine diphosphate glucose; Hexose-P, hexose phosphate; G1P, glucose-1-phosphate; ADPG, adenosine 5′-diphosphate glucose; AGPase, adenosine 5′-diphosphate glucose pyrophosphorylase; SS, soluble starch synthase; GBSS, granule-bound starch synthase; SBE, starch branching enzyme; DBE, debranching enzyme; BAM, beta-amylase; AMY, alpha-amylase.

Lily micropropagation *in vitro* is a heterotrophic metabolism process (Gao et al., 2018; Nguyen et al., 2020) in which the shoot and bulblet act as sink organs. As mentioned above, starch is the main storage compound in lily scales. For starch biosynthesis in sink organs, the glucosyl donor is derived from sucrose unloading via the sieve element/companion cell complex (SE/CC complex) in the phloem and further synthesized by the concerted action of several enzymes, including adenosine 5′-diphosphate glucose pyrophosphorylase (AGP), soluble starch synthase (SS), granule-bound starch synthase (GBSS), and starch branching enzyme (SBE). Additionally, debranching enzyme (DBE) is also required to remove some branch points in amylopectin to create a structure that can crystallize. Plant AGP consists of two similar subunits, the small subunit of AGP (AGPS) and the large subunit of AGP (AGPL), which form an active homo-tetrameric enzyme. As for starch degradation direction, alpha-amylase (AMY) and beta-amylase (BAM) are the main players (Harsselaar et al., 2017; Smith and Zeeman, 2020). Sucrose is widely used as the primary and sole carbon source in lily tissue culture media (Gao et al., 2018; Nguyen et al., 2020). Normally, sucrose is unloaded from the phloem into sink cells either apoplastically or symplastically. For the former, sucrose is transported by plasma membrane-localized sugars will eventually be exported transporters (SWEET) and broken down into fructose and glucose by cell wall invertase (CWIN) before being taken up into cytoplasm. For the latter, sucrose is exported by plasmodesmata (PDs) (Chen et al., 2012; Ruan, 2014; Fernie et al., 2020) (Figure 1). Additionally, it was recently reported that sucrose not only is the main carbon supply but also is likely to play a role in signaling during *in vitro* lily bulblet formation (Gao et al., 2018). However, the molecular mechanisms involved in starch and sucrose metabolism during bulblet formation *in vitro* are still poorly understood.

The growth regulator forchlorfenuron [*N*-(2-chloro-4-pyridyl)-*N*′-phenylurea (CPPU)] is a highly active cytokinin-like phytohormone; its biological activity is 10 to 100 times higher than that of 6-benzylaminopurine (Zeng et al., 2016). CPPU is an example of a synthetic cytokinin that can regulate cell division and organogenesis (Mihaljević and Vršek, 2009; Subotić et al., 2009) as well as fruit set and development (Lewis et al., 1996; Zeng et al., 2016), parthenocarpy (Zhang and Whiting, 2013; Hu et al., 2019), fruit shelf life (Huang and Jiang, 2012), and abiotic stress resistance (Gashaw et al., 2014). Most of these processes have been investigated in fruit and vegetable crops. Conversely, the application of CPPU in flower bulbs is rather limited. Several reports have shown that various CPPU concentrations have beneficial effects on bulblet enlargement in *Lycoris* species, suggesting that the effect is genotype dependent (Xiao et al., 2013; She et al., 2014; Ren et al., 2017a). Moreover, the favorable effects were attributed to higher starch accumulation in the bulblets and the enhanced activity of starch metabolism-related enzymes. However, the effects of exogenously applied CPPU on lily bulblet formation have not been reported.

In this study, we showed that the exogenous application of a high concentration of CPPU prevents the shoot-to-bulblet transition in ‘Sorbonne’ lily. Therefore, we compared the responses to bulblet-inducing and bulblet-non-inducing conditions at the morphological, histological, and physiological levels. To better understand starch-related gene expression, a comprehensive description of the genes involved in starch metabolism was performed based on our PacBio and Illumina transcriptome database. Two genes encoding key starch synthesis enzymes were cloned and phylogenetically analyzed. Moreover, we also explored gene transcriptional levels related to starch and sucrose metabolism. A preliminary fluorescent tracer technique using 6(5)-carboxyfluorescein diacetate (CFDA) was used to monitor sucrose unloading. This study aims to explore the factors influencing starch–sucrose metabolism under bulblet-inducing and bulblet-non-inducing conditions in lily to provide mechanistic insights into the control of bulblet formation.

## Materials and Methods

### Plant Materials, Growth Conditions, Design, and Sampling

The scales of ‘Sorbonne’ bulbs (16–18 cm in circumference) purchased from Zhejiang Hongyue Seed Co., Ltd., China, were used as the starting explants. Individual shoots were obtained as mentioned in a previous protocol (Wu et al., 2017). The cultures were incubated at 24 ± 2°C under cool white fluorescent tubes emitting 60 μmol photons m^–2^s^–1^, with a 12:12-h light:dark photoperiod at the Physiology & Molecular Biology Laboratory of Ornamental Plants and Tissue Culture Laboratory of Ornamental Plants at Zijingang Campus, Zhejiang University, China (E 120°11′, N 30°29′).

The experiments were performed in 2013 and 2016–2017. The shoots were adopted as primary explants in our experiments and subsequently transferred to basal Murashige and Skoog (MS) medium (Murashige and Skoog, 1962) containing 8% agar and 0.2 M of sucrose with various concentrations of PGRs including 5 × 10^–2^ mM of CPPU, 5 × 10^–4^ mM of PBZ, and control (CON) without any exogenously applied PGRs. A total of 200 shoots were used for each treatment, and each treatment had three replicates. Five shoots were transplanted in each glass conical flask (6.5-cm diameter × 10-cm height). The experiments were conducted in a completely randomized design. CPPU and PBZ were added to the medium directly before autoclaving. Random plantlets from each treatment were harvested during different bulblet developmental stages at 9:00 a.m. (Beijing time). The specific sampling dates for different experimental traits are listed in Supplementary Table 1.

### Morphological and Histological Analysis

Several morphological indices, including plantlet height (PH), number of leaves (NL), number of roots (NR), root length (RL), fresh bulblet weight (FBW), fresh plantlet weight (FPW), and bulblet diameter (BD), were determined at defined sampling dates (Supplementary Table 1). Specifically, 0 days after transplanting (DAT) represented the start time, and the default values for PH, NL, NR, RL, FBW, and FPW were 1.00 cm, 0 mg, 0 mg, 0 mg, 0 mg, and 50 mg, respectively.

**FIGURE 2:**
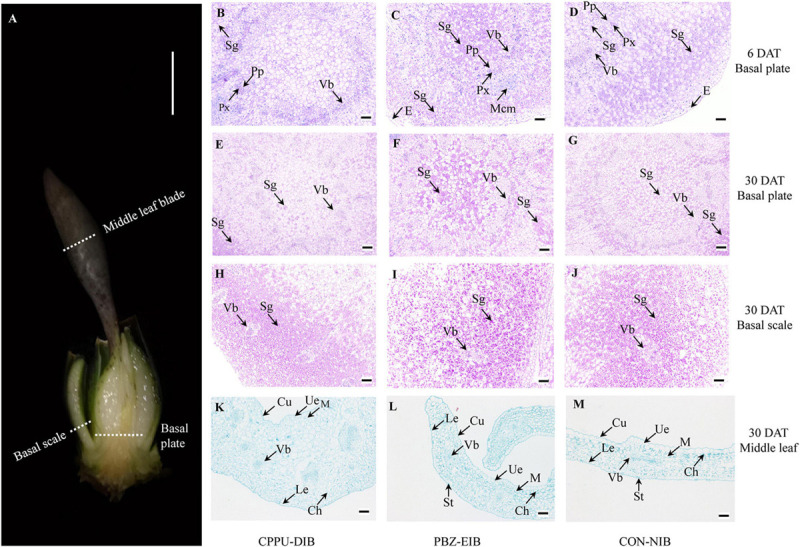
Cytological observations. **(A)** Sampling site. **(B–G)** Periodic acid-Schiff staining in basal plate. (**H–J**) Periodic acid-Schiff staining in basal scale. **(K–M)** Safranin O-Fast Green staining in middle leaf blade. White scale bar = 0.5 cm; black scale bar = 100 μm. DAT, days after transplanting; CPPU-DIB, forchlorfenuron defective in bulbification; PBZ-EIB, paclobutrazol enlargement in bulbification; CON-NIB, control-normal in bulbification; Sg, starch grain; Vb, vascular bundle; Px, primary xylem; Pp, primary phloem; Mcm, meristem cell mass; E, epidermis; Cu, cuticle; Ue, upper epidermis; Le, lower epidermis; Ch, chloroplast; St, stoma; M, mesophyll.

To visualize the starch content and observe the distinctive bulblets/shoots cytologically, basal plates and basal scales of samples (Figure 2A) from specific developmental stage (before bulbification at 6 DAT, and bulblet filling stage at 30 DAT) of three treatments were examined using the modified periodic acid-Schiff (PAS) method of Mowry (1963) as described by Wu et al. (2017). The tissues were fixed in FAA fixation buffer (formaldehyde:glacial acetic acid:ethanol, 1:1:18, v/v) for 24 h and embedded in paraffin, and then 12 μm semi-thin sections were cut with a microtome (Leica 2016, Leica Microsystems, Heidelberg, Germany). The sections were oxidized for 10 min in periodic acid, treated with Schiff’s reagent for 15 min, and then stained with hematoxylin for 2 min. The middle leaf blade (Figure 2A) samples were stained with 1% (w/v) Safranin T and 0.5% (w/v) Fast Green FCF (Yang et al., 2017). The PAS kit (G1008) was purchased from Wuhan Goodbio Technology Co., Ltd. (Wuhan, China). The sections were examined under a Nikon ECLIPSE CI microscope (Nikon Corp., Tokyo, Japan) and photographed with a Nikon DS-U3 digital camera connected to the microscope. In PAS staining, the polysaccharide, e.g., starch, is mauve, and the nucleus is blue.

### Non-structural Carbohydrate Content Assay

Non-structural carbohydrates, i.e., sucrose, total soluble sugar, and starch, were extracted from approximately 0.5 g [fresh weight (FW)] samples of bulblets. Extractions were performed as previously described (Wu et al., 2019a). The liquid supernatants were combined and used for the estimation of sucrose and total soluble sugar and were determined by the modified anthrone method (DuBois et al., 1956). The remaining pellets were re-extracted for starch determination in accordance with the procedure of McCready et al. (1950). All final samples (200 μl) were arranged in a 96-well enzyme label plate (Costar, 3590, United States), and a spectrophotometric analysis was conducted with a Multilabel Reader (Perkin Elmer Corporation, Enspire^TM^ 2300, United States). The absorbance of the supernatant was read at 620 nm.

### Genome Size Estimation

Nuclei were prepared by sampling 50–100 mg of fresh young leaves of ‘Sorbonne’ in extraction buffer as previously published (Dolezel et al., 2007). Each leaf sample was cut with a razor blade and incubated for 10 min on ice. The homogenate was filtered through a 30-μm mesh filter and mixed with 50 μg/ml of propidium. Flow cytometry measurement was taken using BD FACSCalibur^TM^ platform (Becton Dickinson, San Jose, CA, United States) equipped with a 488-nm laser canon. *Ginkgo biloba* (1C = 10.61 Gb) was used as an internal standard (Guan et al., 2016). To estimate the genome size of the investigated taxa, three individuals were analyzed. A DNA content of 10,000 stained nuclei was determined for each sample. The relative fluorescence histograms were analyzed on ModFit software (Version 2.3). ‘Sorbonne’ is diploid as well (2*n* = 2*x* = 24) (Li et al., 2011). Based on the peak of internal standard and ‘Sorbonne’, the experimental genome size was calculated according to the following equation: sample genome size = (sample G1/standard G1) × standard genome size.

### Identification of Genes Encoding Starch Metabolism-Relevant Enzymes

For the identification of gene-coding enzymes involved in starch metabolism, two reports on *Arabidopsis thaliana* (Sonnewald and Kossmann, 2013) and *Solanum tuberosum* (Harsselaar et al., 2017) were used as the starting point. Three previously assembled and annotated transcriptomes for ‘Sorbonne’ in our group were used to comprehensively analyze major starch metabolism-related genes. The primary merged transcriptome is for three different treatments in the current study using the *in vitro* bulblets (60 DAT) as the sequencing material (GenBank accession number, SRR1390677). The second transcriptome is from a mixture of six tissues including basal roots, scales, leaves, stem epidermis, tepals, and stigmas (GenBank accession number, SRP019507). The third transcriptome is based on the Pacbio sequencing platform (unreleased one). Keyword searches for each annotation file were conducted, and the genes annotated to the same *Arabidopsis* gene number were filtered by the longest predicted open reading frame (ORF) using TBtools (Chen et al., 2020). Moreover, we searched the released starch-related genes in *Lilium* genus by BLASTp one by one.

### RNA Isolation

Total RNA was isolated from different tissues (leaf, stem, bulblet, and root) or bulblets of different developmental stages using the EASYspin Plus Plant RNA kit (RN38, Aidlab Bio, Beijing, China) according to the manufacturer’s protocol, which was followed by DNaseI treatment (4716728001, Roche, Basel, Switzerland). RNA integrity was verified on 1% agarose gel. RNA purity and concentration were estimated by ultraviolet spectroscopy with a NanoDrop 2000 spectrophotometer (Thermo Scientific, Madison, WI, United Sattes).

### Gene Cloning, Sequence, and Phylogenetic Analysis

The *AGPS* and *SSS* partial gene fragments were obtained from the transcript of previously sequenced data of *in vitro* ‘Sorbonne’ bulblet. To obtain the complete cDNAs of the target genes, rapid amplification of cDNA ends (RACE) technique was used in this study. cDNA synthesis was performed according to SMARTer^TM^ RACE cDNA Amplification Kit (634858, Clontech, Mountain View, CA, United States). Universal primers and adaptor primers were provided by the kit. All the gene-specific primers were designed by Beacon Designer 7 and are listed in Supplementary Table 2. Primers were synthesized by Sangon Biotech (Shanghai, China). Nested PCR was used to obtain the 5′ and 3′ ends of the cDNAs. The 5′ and 3′ RACE-PCR products were purified using a gel extraction kit (740609, MN, Düren, Germany) and subcloned by insertion into a pGEM-T easy Vector (A1360, Promega, Madison, WI, United States) and transferred into *Escherichia coli* cells (DH5α). Then, at least three positive clones from ampicillin-containing media were selected and sequenced by Sangon Biotech.

The ORFs of LohAGPS and LohSS were confirmed by BLASTp and ORF Finder^1^. The molecular weight (MW) and theoretical isoelectric point (pI) were predicted by using an ExPASy analysis system^2^. The membrane protein was predicted by TMHMM 2.0^3^ (Krogh et al., 2001). The 3D structural model of the protein is predicted by homology modeling with SWISS-MODE^4^ (Kopp et al., 2005). The subcellular localization of the deduced polypeptides was predicted by Cell-Ploc 2.0^5^ (Chou and Shen, 2010). The multiple sequence alignment of the proteins with their homologs from other plant species and the calculation of identity were conducted using MAFFT software (v7.305b) (Kazutaka and Standley, 2013). Protein sequences of the *AGP* and *SS* genes in other species were downloaded from the PLAZA database (Version 4.5^6^) and the National Center for Biotechnology Information (NCBI) database^7^. All the sequences (Supplementary Tables 3, 4) were aligned using Clustal-X1.8 (Thompson et al., 1997), and the phylogenetic trees were subsequently constructed in FastTree 2.1 (Price et al., 2010) to compute approximately maximum-likelihood trees. The tree was collapsed and formatted using iTOL v4 (Letunic and Bork, 2019).

### cDNA Synthesis and Quantitative Real-Time RT-PCR Analysis for Selected Key Genes

cDNA was reverse-transcribed using M-MLV reverse transcriptase according to the manufacturer’s instruction (M5313, Promega, Madison, WI, United States). qRT-PCR was used to detect the relative expression levels of a selected set of genes. Primers for the qRT-PCR analysis were designed using Beacon Designer 7 based on the lily transcriptome database. Based on a previous bulblet development biological process study in *Lilium davidii* var. *unicolor* (Li et al., 2014) and our preliminary experiment for evaluation of candidate reference genes, *GAPDH* (Primer-F: GAATGGCAAGCTAACTGGAATG; Primer-R: CAGCCTTGATCTGATCGTAAGT) was used as an internal control. qRT-PCRs were performed with TB Green^TM^ Premix Ex Taq^TM^ Kit (RR420A, TaKaRa, Tokyo, Japan) in a Bio-Rad Connect^TM^ Optics Module (Bio-Rad, Hercules, CA, United States). The reaction volume was 20 μl with 10 μl of TB Green^TM^ Premix Ex Taq^TM^ (2×), 0.8 μl of each forward and reverse primer (10 μM), 5 μl of cDNA, and 3.4 μl of PCR-grade water. The cycle conditions were as follows: 95°C for 3 min, 40 cycles of 95°C for 30 s, 55°C for 30 s, and 72°C for 1 min. Three RNA isolations and triplicate qRT-PCR runs were implemented for each sample for biological and technical replication. Relative quantitation (RQ) was calculated using the 2^–ΔΔCt^ method, after normalization based on the reference gene (Livak and Schmittgen, 2001). Tissue-specific expression was performed only for two cloned genes. Other major starch and sucrose metabolism genes expression was measured for different developmental stages under three treatments. All the primers used for qRT-PCR are listed in Supplementary Table 5. Moreover, all the qRT-PCR examined genes were screened from the RNA-Seq database, and the gene expression levels were evaluated according to the fragments per kilobase of exon per million mapped reads (FPKM) values. The expression patterns of these genes were illustrated using a heatmap by TBtools (Chen et al., 2020) and compared with qRT-PCR data.

### Labeling of 6(5)-Carboxyfluorescein Diacetate and Observations

To determine the phloem unloading pathway, we applied fluorescent tracer CFDA to characterize the transport pathway as described in Roberts et al. (1997) and Wu et al. (2012) with modification (Supplementary Figure 1). Double-edged prep blades were used to produce a wedge-shaped block in the middle-upper part of the abaxial surface of the mother bulblet scale or the outermost scale of newly formed bulblet to expose the major vascular bundle, and then 120 μl of CFDA (1 mg/ml, C4995, APExBIO, Houston, United States) was imported into a pledget-filled block with an Eppendorf pipette and was then covered with polythene film and aluminum foil to prevent evaporation and degradation of the dye. The CFDA-treated materials were inoculated back to the original growing medium to allow the carboxyfluorescein (CF) to be transported for at least 24 h. All the manipulations were carried out in a laminar flow cabinet with sterile material. The basal part of the sink organs, including the shoot and scale adjacent to the CFDA-imported material, was fixed in Tissue-TeK^®^ O.C.T. compound (Sakura Finetek, Torrance, CA, United States) and was crosscut with a Shandon Cryotome FE (Thermo Scientific, Madison, WI, United States). The ultrathin sections were photographed under the blue light of a fluorescence microscope (Eclipse Ni, Nikon, Tokyo, Japan).

### Statistical Analysis and Illustration Creation

All morphological traits consisted of at least 10 biological replicates, and all other measurements consisted of three biological replicates unless indicated otherwise. Statistical analyses were conducted based on a one-way analysis of variance in IBM SPSS Statistics 20.0 software (IBM, Inc., Armonk, NY, United States) with Duncan’s multiple range test. The calculation of relative bulblet weight (RBW) and bulblet size (BS) was described in our previous study (Wu et al., 2019a). The shoot-to-bulblet transition rate refers to the ratio of bulblet number and plantlet number. The ratio of parenchyma cells (PCs) with a visible nucleus equals the number of PC containing visible nucleus/100 PCs. The 100 PCs were those that were close to the vascular bundle system in the basal plate. The ratio of PCs with starch grains is equal to the number of PCs containing a starch grain/100 PCs. The 100 PCs were those that were located inside the circular vascular system in the basal plate. The relative starch grain size was determined using the grain area values measured by ImageJ divided by the smallest one. Illustrations were drawn using GraphPad Prism 8 and PowerPoint software (Microsoft Office 365 ProPlus).

## Results

### High Levels of *N*-(2-Chloro-4-Pyridyl)-*N*′-Phenylurea Prevented Bulblet Formation

**FIGURE 3:**
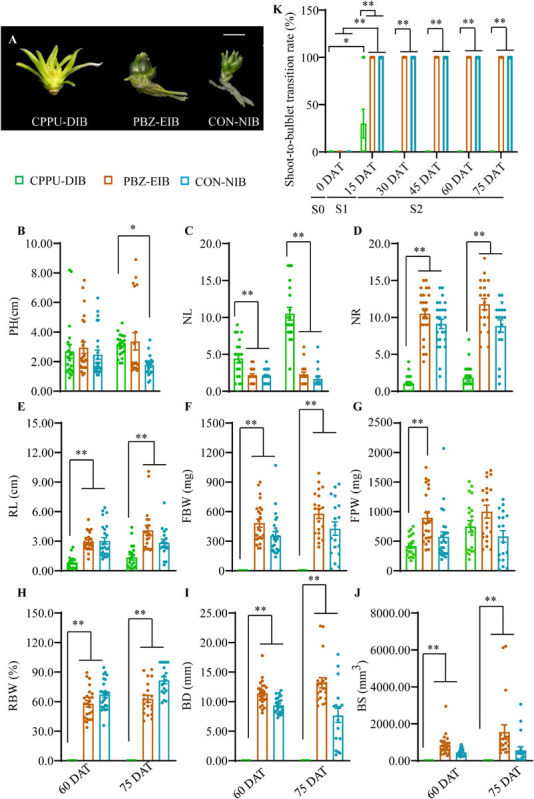
Growth status in different treatments. **(A)** Phenotypes recorded at 60 DAT in the experimental year 2016. **(B–J)** Morphological trait differences at later developmental stages. Data were based on two experimental years, and the solid circles are biological replicate data (*n* = 25). The central values represent the means, and the error bars indicate the SEM of all biological replicates. PH, plantlet height; NL, number of leaves; NR, number of roots; RL, root length; FBW, fresh bulblet weight; FPW, fresh plantlet weight; RBW, relative bulblet weight; BD, bulblet diameter; BS, bulblet size. RBW refers to the ratio of FBW and FPW. Radius (*r*) was derived from the BD. Inputting the value of *r* in the formula 4/3π*r*^3^, the bulblet size (BS) was calculated. **(K)** Changes in shoot-to-bulblet transition rate in the experimental year 2013. Shoot-to-bulblet transition rate refers to the ratio of bulblet number and plantlet number. Only outer leaf blades were scale-like for CPPU-DIB at 15 DAT, and they were recorded as bulblets. Data are represented as the means ± SEM (*n* = 10 biological replicates). S0 (0 DAT), the initial shoot stage; S1 (0–15 DAT), the key shoot-to-bulblet transition stage; S2 (15–75 DAT), late plantlet developmental stage. DAT, days after transplanting; CPPU-DIB, forchlorfenuron defective in bulbification; PBZ-EIB, paclobutrazol enlargement in bulbification; CON-NIB, control normal in bulbification. Asterisks indicate significant differences (Duncan’s multiple range test, ^∗^*p* < 0.05, ^∗∗^*p* < 0.01).

A 2-year *in vitro* experiment was conducted under various bulblet-inducing conditions (Figure 3A). The observed differences in morphology under the different conditions included a lack of bulbification with the CPPU treatment (named CPPU-DIB; also referred to as defective in bulbification) and an enlargement in bulbification with the PBZ treatment (named PBZ-EIB) and the control treatment (named CON-NIB; NIB is short for normal in bulbification). Linear leaf whitening was observed in CPPU-DIB, whereas green lanceolate leaves were observed in CON-NIB and PBZ-EIB (Figure 3A and Supplementary Figure 2A). For the four main shoot and root indices, CPPU-DIB showed inhibition in the roots only. More specifically, PH was consistently lower than 3 cm during the whole growth process in 2013, with a 2-year average PH of 2.70 cm at 60 DAT. Nonetheless, no significant difference in PH was observed among the all three treatments at this time point. CPPU-DIB showed an increase in NL, and the highest 2-year average number was 10.5. The suppression of root growth was severe in terms of both NR and RL (Figures 3B–E and Supplementary Figures 2B–E). The same root growth inhibition effect was also observed *in vitro* in the two treatments along with a lower CPPU concentration (data not shown), indicating that exogenously applied CPPU hindered lily root growth. FBW showed only positive values at the very beginning of bulblet initiation (15 DAT) but declined to 0 afterward. Meanwhile, FPW climbed gradually during growth. Thus, RBW measurements showed a similar trend as the FBW (Figures 3F–H and Supplementary Figures 2F–H). Accordingly, the BD and BS for CPPU treatment were 0 at 60 DAT and after as no bulblets formed (Figures 3I,J).

Intriguingly, we noticed that under bulblet-inducing conditions, the shoot-to-bulblet transition rate rapidly reached 100% at 15 DAT (Figure 3K). In fact, the shoot showed a very quick visible response: the outermost leaf sheath darkened and thickened by ∼7–8 DAT and formed the original outer scale of the bulblet, implying that in successful shoot-to-bulblet transitions, the stage between 0 and 15 DAT is crucial for determining the fate of the bulblet determination. Meanwhile, the roots were produced almost concurrently with the bulblet in these treatments to absorb the nutrient and carbon sources from the medium. Nevertheless, under bulblet-non-inducing condition (CPPU-DIB), the outer scale-like tissue emerged only temporarily and partially (30%) at 15 DAT and reverted to shoot tissue afterward (Figure 3K). Accordingly, we divided the whole developmental process into three stages: S0 (0 DAT), which is the initial shoot stage; S1 (0–15 DAT), which is the key shoot-to-bulblet transition stage; and S2 (15–75 DAT), which is the late plantlet developmental stage (Figure 3K).

### Cytological Observations Revealed Abnormal Starch Allocation and Undeveloped Leaf Blades in the DIB Treatment

**FIGURE 4:**
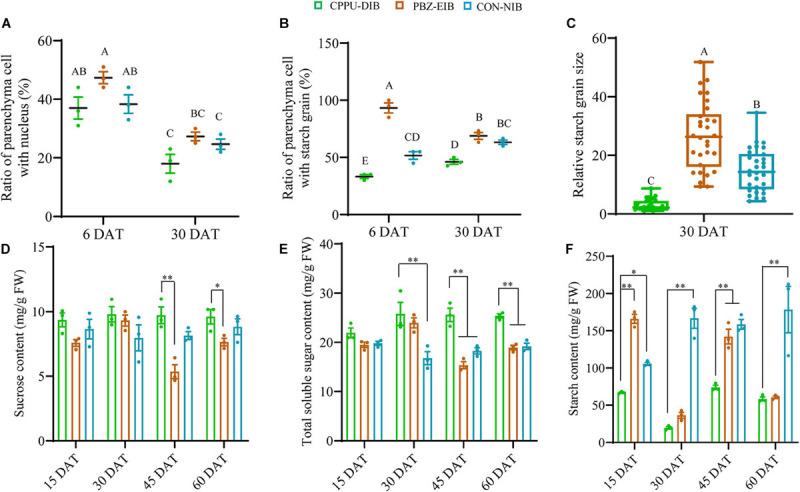
Major non-structural carbohydrates. **(A)** The ratio of parenchyma cells (PCs) with a visible nucleus in the basal plate. The counting was based on 100 PCs around a vascular bundle. The ratio refers to the number of PCs with visible nucleus (blue particle)/100 PCs. Data are represented as the means ± SEM (*n* = 10 biological replicates). **(B)** The ratio of PCs with starch grains in the basal plate. The counting was based on 100 PCs inside the circular vascular system. Data are represented as the means ± SEM (*n* = 10 biological replicates). **(C)** Relative starch grain size in the basal scale. All area values measured by ImageJ were divided by the smallest one to derive the relative size. Data are represented as box plot ± SEM (*n* = 30 biological replicates). Different uppercase letters indicate significant differences at *p* < 0.01 according to Duncan’s multiple range test. **(D–F)** Content of non-structural carbohydrates. The solid circles are biological replicate data (*n* = 3). The central values represent the means and the error bars indicate the SEM of all biological replicates. DAT, days after transplanting; CPPU-DIB, forchlorfenuron defective in bulbification; PBZ-EIB, paclobutrazol enlargement in bulbification; CON-NIB, control normal in bulbification. Asterisks indicate significant differences within each column (Duncan’s multiple range test, ^∗^*p* < 0.05, ^∗∗^*p* < 0.01).

To clarify the differences among these three treatment responses from a cytological perspective, systematic observations were carried out on the scale, basal plates, and leaves. The specific sampling site is shown in Figure 4A. The basal plate indicates the compressed stem, which is analogous to a flattened plate from which roots are produced and to which modified imbricate fleshy leaves (scales) are attached. It is not easy to separate, extract, and measure the non-structural carbohydrate contents in this part. Histological observations are therefore helpful to visualize the polysaccharide (mainly starch) status. The basal scale in the current study refers to the basal part of the outermost scale. The lateral section of the basal plate at an early stage (6 DAT, before bulbification) showed that considerable numbers of vascular bundles were distributed evenly in the basal plate, forming a circular vascular system (Supplementary Figure 3). Additionally, this area is rich in nuclei and demonstrates active cell differentiation. However, no significant difference was observed in the ratio of PCs with a visible nucleus index among all treatments. The major distinction between bulblet-non-inducing and bulblet-inducing conditions was the amount of starch grains near the vascular system. In the CPPU-DIB treatment, few starch grains were inside or close to the circular vascular bundles, and more starch grains were near the epidermis. However, the PBZ-EIB treatment had the highest abundance of small starch grains inside the vascular system, while the CON-NIB treatment had slightly fewer starch grains that were also inside the vascular system (Figures 2B–D, 4A). As bulblet development progressed, the proportion of nuclei declined dramatically for all treatments. Nevertheless, the percentage of PCs containing starch grains decreased significantly for PBZ-EIB and increased slightly for CON-NIB, but the difference was not significant; the ratio of PCs with starch grains inside the basal plate increased significantly in bulblet-non-inducing treatment, indicating a time lag in starch accumulation within the basal plate (Figures 2E–G, 4B). At the later bulblet filling stage (30 DAT), much larger starch grains accumulated in the outer scale in the bulblet-inducing treatments than in the DIB treatment (Figures 2H–J, 4C). To understand the condition of possible source, we also observed the leaf blade anatomical structure. The major features observed in bulblet-non-inducing treatment were fewer chloroplasts in the mesophyll cell, fewer stomata on the lower epidermis, and a more regular arrangement of mesophyll cells (Figure 2K). In contrast, under bulblet-inducing conditions, more chloroplasts and stomata and loosely arranged mesophyll cells were observed (Figures 2L,M).

### *N*-(2-Chloro-4-Pyridyl)-*N*′-Phenylurea–DIB Treatment Accumulated Much Less Starch but More Sugars

By measuring the non-structural carbohydrate content, we found that the bulblet-non-inducing treatment accumulated more sucrose at a steady level during the whole growth period. Conversely, the bulblet-inducing treatment, PBZ-EIB, resulted in significant fluctuations in sucrose content, especially at the later filling stages (30 DAT and afterward) (Figure 4D). A clearer trend was observed for the total soluble sugar content; CPPU-DIB was observed to have a significantly higher total soluble sugar content than both bulblet-inducing treatments at 45 and 60 DAT (Figure 4E). Nevertheless, the starch content of CPPU-DIB was significantly lower than that of the bulblet-inducing treatments at 30 and 60 DAT except PBZ-EIB, and the highest starch content was 73.85 mg/g FW observed in CPPU-DIB (Figure 4F).

### Screening for Genes Involved in Starch Metabolism Based on Our Previous Transcriptome Data

Starch is the principal component of the lily bulblet. However, a complete inventory of genes involved in starch metabolism has not yet been performed due to the large genome and lack of genome sequences in *Lilium*. The genome size for ‘Sorbonne’ calculated based on flow cytometry was 37.98 ± 0.48 Gb (Supplementary Figure 4A), which was slightly smaller than that of its parent species *Lilium auratum* (55.44 ± 10.18 Gb) (Du et al., 2017b). By using two model plants, *A. thaliana* and *S. tuberosum*, as references, we fully explored the ‘Sorbonne’ transcriptome data in our research group. Based on sequence similarity, 52 out of 58 *Arabidopsis* ORFs were assigned to homologous ‘Sorbonne’ transcripts. No homologous sequences were found for At4g39210 [Large subunit of ADP-Glucose Pyrophosphorylase 3 (APL3)], At2g21590 [Large subunit of ADP-Glucose Pyrophosphorylase 4 (APL4)], At1g05610 [Small subunit of ADP-Glucose Pyrophosphorylase (APS2)], At2g32290 [beta-amylase 6 (BAM6)], At5g65685 [Starch synthase 5 (SS5)], and At5g17310 [UDP-glucose pyrophosphorylase 2 (UGP2)] in any of the assembled data. After prediction with software and manual filtration, the longest ORFs for each transcript were listed, and their completeness was estimated according to the relevant protein length of *A. thaliana*. Most of the ORFs were regarded as complete coding sequences (Supplementary Table 6). We focused on AGP and SS, and the identified ORFs were selected and listed. Specifically, a total of nine unique LohAGPs originating from 58 assembled unigenes (isoforms) and 19 for LohSSs from 88 unigenes (isoforms) were identified from our transcriptome data. Eventually, the predicted ORF sequences of all identified genes were compared with published mRNA sequences available on the NCBI database via a BLASTp search. Despite the crucial importance of starch metabolism, only 10 records were found, from a total of four lily genotypes (*Lilium brownii* var. *viridulum*, ‘Siberia,’ ‘Sorbonne’, and *Lilium davidii* var. *unicolor*), indicating the insufficiency of *Lilium* genomic resources. A comparison of the sequence similarity revealed that the identity scores were more than 97.4% for the other three lilies, which suggests the data provided in the present study will be highly useful for future studies.

### Characterization of LohAPS and LohSSIII

AGP and SS are key enzymes that catalyze the main steps of starch synthesis. To confirm the reliability of the transcriptomically derived ORFs, AGP and SS were selected for validation. After sequencing and the assembly of fragments, full-length cDNAs of *APS* and *SS* were acquired and deposited in the GenBank (accession Number KX398951, designated *LohAGPS*; MF101406, designated *LohSS*). The complete sequence of *LohAGPS* was 1,929 bp, and it contained an ORF of 1,569 bp encoding 522 amino acids. The full length of *LohSS* was 4,074 bp, and it contained a complete ORF of 3,594 bp encoding 1,197 amino acids. The cloned AGPS protein sequence was completely consistent with the ORF derived from Isoform_31988, whereas the SS sequence exhibited sequence differences from any filtered ORFs based on the transcriptomic data. After phylogenetic analysis, LohAGPS and LohSS were named LohAGPS1.2b and LohSSIIId. In terms of the two experimental cloned isoforms, the relative MWs of LohAGPS1.2b and LohSSIIId were 56.85 and 131.48 kDa, respectively. Furthermore, both of these proteins were located in the chloroplast (Supplementary Figure 4B). The alignment of the AGPS protein sequences from different plant sources showed that LohAGPS was weakly conserved in the N-terminus and that most of the similarities were concentrated in the following putative conserved regions: ATP-binding site (WFQGTADAV), catalytic site (LAGDHLYRMDY), Glc-1-phosphate binding site (IIEFAEKPKGE), and 3-phosphoglycerate activator site (SGIVTIIKDALIPSGTVI) with the conserved NTP domain (PF00483) (Supplementary Figure 4C and Supplementary File 1) (Cheng et al., 2015). LohSS harbored both a Glyco_transf_1 (PF00534) domain and a Glyco_transf_5 (PF08323) domain in the C-terminal region and three CBM_25 domains in the middle (Supplementary Figure 4D) (Yang et al., 2013) but had a significantly variable N-terminal region (Supplementary Figure 4B).

**FIGURE 5:**
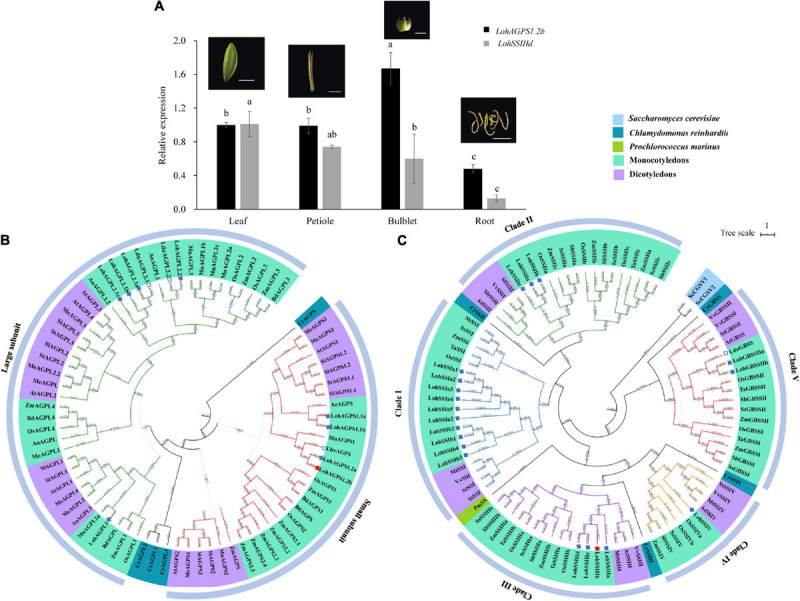
Tissue-specific expression pattern and the phylogenetic analysis of *LohAGPS1.2b* and *LohSSIIId*. **(A)** Gene expression in different tissue. Data are represented as the means ± SEM (*n* = 3 biological replicates). The different lowercase letters indicate significant difference at *p* < 0.05 according to Duncan’s multiple range test within each gene. **(B,C)** Phylogenetic tree of AGPase and starch synthases in different plants by FastTree software. The genes from *Lilium* Oriental hybrids in the present study and other *Lilium* species from National Center for Biotechnology Information (NCBI) are marked by solid and hollow diamonds, respectively. The solid red diamond indicates the verified gene by rapid amplification of cDNA ends cloning.

To investigate the tissue expression pattern, we harvested samples of four different tissues (leaf, petiole, bulblet, and root) from the tissue culture-derived plantlet. Although *LohAGPS1.2b* and *LohSSIIId* were both expressed in the four assayed tissues, differential expression levels were observed. The highest relative expression of *LohAGPS1.2b* was shown in the bulblet, followed by the petiole, leaf, and root. However, the expression of *LohSSIIId* seemed to be higher in leaves and bulblets than in petioles (Figure 5A).

### Phylogenetic Tree of the LohAGPs and LohSSs

A total of 77 AGPs were used to construct the phylogenetic relationships. These AGPs were divided into two groups: a small-subunit group and a large-subunit group. The small-subunit group contained 30 AGPs, and the large-subunit group contained 43 AGPs. For ‘Sorbonne,’ the results showed that two LohAGP large subunits and one LohAGP small subunit were classified: LohAGPL1, LohAGPL2, and LohAGPS1. LohAGPL2 had high similarity to two AGPLs from *L. davidii* var. *unicolor*, whereas LohAGPS1.2 had the highest similarity to an AGPS from *L. brownii* var. *viridulum*. Additionally, all the sequences could be divided into two major subclades, i.e., monocots and dicots, with one exception from *Zea mays* (Zm00001d045367). All AGPs from lily clustered within the monocot clade, which is consistent with its taxonomic grouping (Figure 5B).

To identify phylogenetic inferences for the SSs among the representative species, 90 SS sequences from six monocots, four dicots, and three outgroup species were used to build a phylogenetic tree. Unlike the AGPs, the SSs had five isoforms, and the cloned SS in the present study was in the SSIII clade. Overall, different numbers of LohSSs were classified into paralogous clades, while clade I consisted of mostly homologs. Compared with the AGPs, only one GBSS protein from *L. davidii* var. *unicolor* was found, and it showed a close distance to LohGBSSII, with an alignment similarity of up to 97.4% (Figure 5C).

### Starch–Sucrose Metabolism-Related Gene Expression During the Shoot-to-Bulblet Transition

**FIGURE 6:**
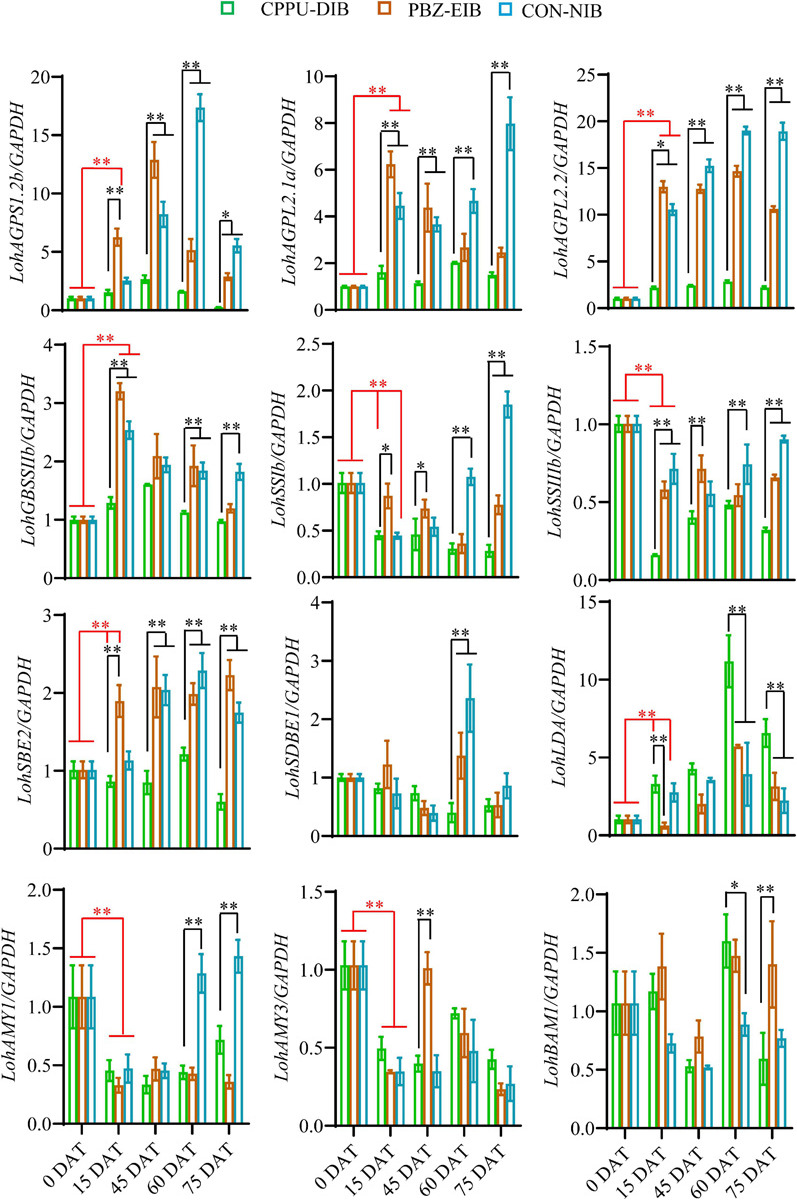
Relative expression of starch metabolism-related genes in different phenotypes during developmental stages. Data are represented as the means ± SEM (*n* = 3 biological replicates). DAT, days after transplanting; CPPU-DIB, forchlorfenuron defective in bulbification; PBZ-EIB, paclobutrazol enlargement in bulbification; CON-NIB, control normal in bulbification. Asterisks indicate significant differences between column (red) or within each column (black) (Duncan’s multiple range test, ^∗^*p* < 0.05, ^∗∗^*p* < 0.01).

The mRNA expression levels of starch-mobilization-related genes were measured using qRT-PCR. Compared with the non-bulbification treatment (CPPU-DIB), the two successful bulblet formation treatments exhibited significantly higher transcript levels of all starch synthesis-related enzymes throughout nearly the whole developmental period, including three AGP genes, one GBSS gene, two SS genes, and one SBE. In addition, the expression of all genes except two SS homologs increased substantially as early as 15 DAT compared with their initial expression levels in shoots (0 DAT) in both the PBZ-EIB and CON-NIB treatments. This finding suggests the dominant role of the starch synthesis pathway during bulbing. For instance, a 13-fold induction in *LohAGPL2.2* transcription was observed at 15 DAT in the PBZ treatment, while only a two-fold increase was observed in the CPPU-DIB treatment. However, the genes involved in starch depletion, including *LohSBE1*, *LohAMY1*, *LohAMY3*, and *LohBAM1*, showed no obvious gene expression response. An increase in expression occurred only at certain developmental time points, which might be ascribed to temporary source-sink modulations in the bulblets under bulblet-inducing conditions. Notably, the CPPU-DIB treatment showed an obvious increase in the gene that encodes Limit dextrinase (*LohLDA*) expression after transplanting, and *LohLDA* expression in the CPPU-DIB treatment was significantly higher at several developmental stages than in the bulblet-inducing treatments (Figure 6).

**FIGURE 7:**
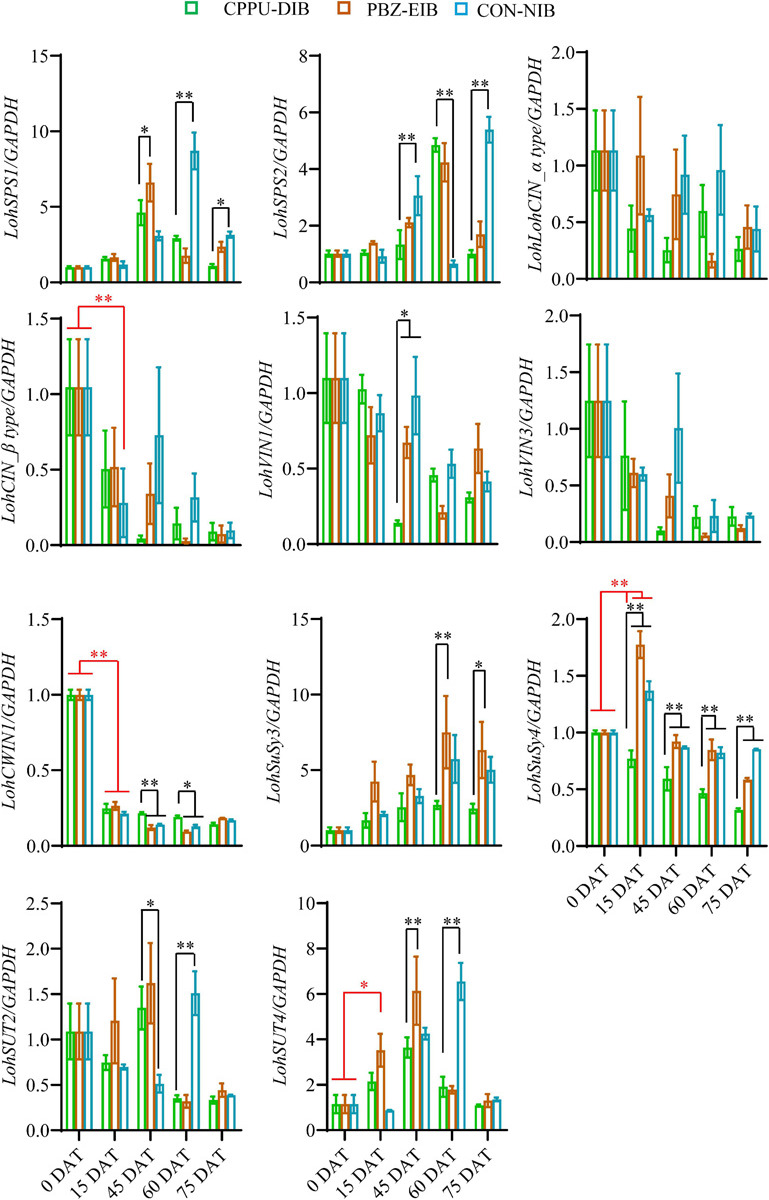
Relative expression of sucrose metabolism-related genes in different phenotypes during developmental stages. Data are represented as the means ± SEM (*n* = 3 biological replicates). DAT, days after transplanting; CPPU-DIB, forchlorfenuron defective in bulbification; PBZ-EIB, paclobutrazol enlargement in bulbification; CON-NIB, control normal in bulbification. Asterisks indicate significant differences within each column (Duncan’s multiple range test, ^∗^*p* < 0.05, ^∗∗^*p* < 0.01).

We further investigated the expression levels of genes involved in the sugar metabolism pathway. There were no clear trends for two sucrose phosphate synthase (SPS) genes tested within the different treatments or at separate developmental stages. Notably, at later growth stages (45 DAT and after), the bulblets exhibited an elevation in mRNA levels to some extent. The gene expression fold changes in cytoplasmic invertase (CIN) and vacuolar invertase (VIN) were minor, and no clear trends were observed. Interestingly, two sucrose degradation enzyme-related genes, namely, *CWIN* and *SuSy*, expressed opposite expression patterns (Figure 7). Thus, *LohCWIN1* expression decreased dramatically from that in the initial shoot (0 DAT), although in the CPPU-DIB treatment, *LohCWIN1* expression was maintained at a significantly higher level than that in the bulblet-inducing treatments at 45 and 60 DAT. Notably, *LohCWIN1* was the only expressed *CWIN* gene in the bulblet scale according to our bulblet initiation process-derived transcriptome data, suggesting its critical role (data not shown). In contrast, the transcript levels of *SuSy* genes in the PBZ-EIB and CON-NIB treatments increased considerably after transplanting and were significantly higher at most developmental stages than in the CPPU-DIB treatment, and *LohSuSy4* was the predominant transcript (the average Ct values for *LohSuSy4* and *LohSuSy3* were 28 and 34, respectively). Moreover, the gene expression patterns of sucrose transporters (SUTs) were similar to those of *LohSPS* in the treatments in the present study (Figure 7).

We obtained the FPKM values of all the qRT-PCR tested genes in our previous RNA-Seq data. Bulblets collected at 60 DAT that were experimental materials in 2013 (Supplementary Table 1) were used in the RNA-Seq. First, the three treatments were divided into two groups in the heatmap: one group was composed of the CPPU-DIB treatment, which is the bulblet-non-inducing group; the other group was composed of the PBZ-EIB and CON-NIB treatments, which is the bulblet-inducing group. In addition, all genes were separated into two main groups based on their expression patterns: one group included genes with relatively high levels in the CPPU-DIB treatment, and the other included genes with the opposite expression trend. Overall, the RNA-Seq data were very similar to our qRT-PCR results (Supplementary Figure 5). Furthermore, we also investigated the transcripts of SWEET, which resulted in 15 hits in the RNA-Seq data. A phylogenetic analysis was carried out after excluding ORFs that were too short, and four clades were established (Supplementary Figure 6A). Within the SWEET gene family, *AtSWEET11* and *AtSWEET12* exclusively transport sucrose during the phloem loading/unloading process (Chen et al., 2015; Jeena et al., 2019). It was noticeable that three transcripts annotated to these two genes were expressed much more in the bulblet-non-inducing treatment than in the other treatments, even at 60 DAT. Moreover, two transcripts annotated to *AtSWEET17*, which is mainly responsible for fructose storage in vacuoles, showed higher expression levels in CPPU-DIB than in the other treatments (Supplementary Figure 6B). This finding might explain the high soluble sugar content observed in the bulblet-non-inducing treatment (Figure 4).

### Observations of Carboxyfluorescein Unloading in Sink Organs

**FIGURE 8:**
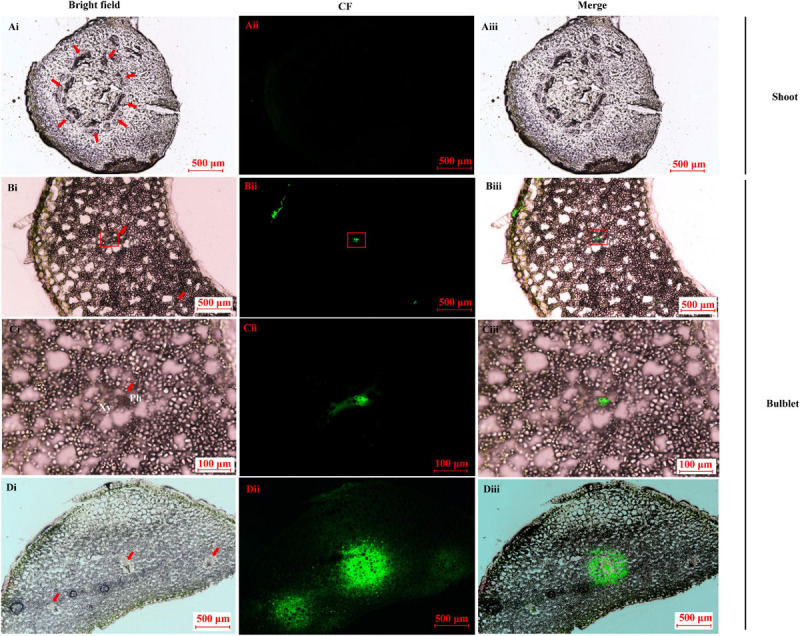
Carboxyfluorescein (CF) fluorescence tracing in the key transition stage of lily *in vitro*. **(A)** Image of CF unloading in shoot basal plate (0 DAT sample) after importing the tracer for 24 h. No CF signal was detected. Bar = 500 μm. **(B–D)** Image of CF unloading in close basal scale after importing the tracer into the outermost bulblet scale (15 DAT sample). The CF was obvious in the vascular bundle (bar = 500 μm) and was restricted to the phloem in the enlarged microscope field **(C)** after 24 h (bar = 100 μm). The CF fluoresce was clearly diffused to the surrounding parenchyma cells, resulting in a bleeding effect at 30 h **(D)**. Bar = 500 μm.

CFDA is a membrane-impermeant fluorescent solute and is a good marker of symplastic phloem unloading (Roberts et al., 1997; Haupt et al., 2001). In the shoot sample before bulbification, no CF was found in any of the vascular systems after CF transporting for 24 h (Figure 8A) and 30 h (data not shown). In contrast, in the newly developed bulblets at 15 DAT, CF was restricted to the internal phloem after 24 h (Figures 8B,C). The extensive spread of CF to the surrounding storage parenchyma elements was evident, and the area around the vascular bundle became highly fluorescent by 30 h (Figure 8D), indicating a symplastic unloading pattern.

## Discussion

The lily bulb is an underground shortened modified petiole. The stem of the bulb forms the bottom of the bulb (the basal plate) with attached scales. In the top of the basal plate, there is an apical meristem, from which aboveground leaves and flowering arrow develop. Plants can be multiplied vegetatively via bulbs, and tissue culture is a main asexual reproduction method for lily, which has significant advantages in shortening the breeding and propagation cycles (Bakhshaie et al., 2016). Bulbing is a critical step during micropropagation, especially for direct organogenesis via shoot formation, which depends heavily on a successful shoot-to-bulblet transition (de Klerk, 2012). Although various external factors that affect the transition process have been studied in *Lilium*, the internal molecular regulatory mechanisms of the transition remain largely unknown. Here, we presented the first systematic report regarding this biological process as it relates to starch–sucrose metabolism.

### Starch Is the Basis of Shoot-to-Bulblet Transition and Further Bulblet Swelling

Research on several crops that have typical storage organs, such as *Allium* and radish, has focused on sucrose metabolism, since sugars (e.g., glucose, fructose, and sucrose) account for most of the dry matter content in these species (Mitsui et al., 2015; Yu et al., 2016; Zhang et al., 2016). However, starch constitutes approximately 70% of the lily bulb based on dry weight; therefore, the starch metabolic pathway is crucial and cannot be ignored. To study such pathways at the molecular level, a sequence analysis of the key genes involved in starch anabolism and catabolism is necessary. First, we performed a comprehensive screening of starch-related genes based on gene enrichment in our transcriptomic data by using *Arabidopsis* and potato as references (Sonnewald and Kossmann, 2013; Harsselaar et al., 2017) (Supplementary Table 6). Two genes, *LohAGPS1.2b* and *LohSSIIId*, were then cloned by RACE, and their sequences were validated (Figure 5, Supplementary Figure 4, and Supplementary Files 1, 2). This work will further increase the current amount of available information about the starch metabolism pathway in *Lilium* since the lily genome is very large and not yet publicly available.

Starch metabolism holds a central position for *in vivo* lily bulbing, for example, in scale cutting system (Li et al., 2014) and in bulbil formation system (Yang et al., 2017). We found that very rapid starch accumulation occurred at the very early stages (15 DAT) in the bulblet-inducing treatments in the current *in vitro* system (Figure 4F). For instance, the starch content in PBZ-EIB remarkably reached 165.9 mg/g FW, which was the highest level during the whole developmental process, while the starch content in the initial shoot was less than 20 mg/g FW (Min et al., 2020). These results indicate that rapid starch enrichment is required for a successful shoot-to-bulblet transition *in vitro*, and starch accumulation can be used as a physiological marker. Indeed, reports have indicated that primordia are plastic and that the transition of a meristem to producing leaf primordia or scale primordia is based on difference of the ability to accumulate starch, according to findings in *Lilium longiflorum* (Bourque et al., 1987). Our cytological observations also supported this hypothesis. In the bulblet-non-inducing treatment, insufficient starch accumulates in the basal plate as early as 6 DAT (Figures 2B–G, 4B), while in the bulblet-inducing treatment, starch in the basal plate is the main carbohydrate pool and translocation compound (Ranwala and Miller, 2008; Wu et al., 2012). In many bulbous species from the Amaryllidaceae family, such as *Lycoris*, *Hippeastrum*, and *Narcissus*, the basal plate is also critical to the successful initiation of new bulblets (Kamenetsky and Okubo, 2012; Ren et al., 2017b). Moreover, the scale in the bulblet-inducing treatment tended to generate long-term storage starch (much bigger) at later stages (30 DAT) than the transitory starch generated in the CPPU-treated plantlet, as determined based on the starch granule size (Figures 2H–J, 4C) (Smith et al., 2005; Wu et al., 2019b). In addition, the number of nuclei showed no difference among the three treatments at 6 DAT and 30 DAT (Figure 4A), indicating the cell proliferation and differentiation are similar within them. The biggest difference should be in the starch number and size resulting in the filling and thickening of the original petiole, which eventually formed the scale. Additionally, the importance of starch is reflected by the significantly elevated expression levels of all starch synthesis genes, including *LohAGPS*, *LohAGPL*, *LohGBSS*, *LohSS*, and *LohSBE*, in the bulblet-inducing treatments in our study (Figure 6 and Supplementary Figure 5). This result supports our hypothesis at the transcriptional level and is in partial agreement with previous results on the bulbil initiation process (Yang et al., 2017), which indicate that it is actually two different biological processes. Overall, we revealed that starch can act as a physiological marker of successful shoot-to-bulblet transition and further bulblet swelling.

### Shoot-to-Bulblet Transition Problems Might Be Associated With the Sucrose Unloading Pathway

We previously confirmed that the starch biosynthesis is essential to bulblet formation and enlargement, which raises the question of how starch synthesis is triggered upstream and what kind of signal cascades are involved. A recent study showed that sucrose acts as signaling molecule instead of an osmotic pressure regulator and carbon source during bulblet induction and expansion in *Lilium sargentiae in vitro*. The exogenous application of sucrose stimulated reactions and signaling in endogenous sucrose (Gao et al., 2018). To this end, we further examined several key genes involved in sucrose metabolism. Surprisingly, we did not find a clear trend in either sucrose synthesis or the tested SUT gene transcript levels. Intriguingly, among the genes encoding sucrose degradation enzymes, we found that *LohCWIN* and *LohSuSy* were differentially expressed during many developmental stages and exhibited nearly opposite expression patterns, especially between different treatments and during the key transition stage (Figure 7 and Supplementary Figure 5). Specifically, *LohCWIN1* expression levels decreased steeply from 0 DAT (the initial shoot stage) to 15 DAT (the visible bulblet stage); relatively high *LohCWIN1* mRNA levels were maintained in the bulblet-non-inducing treatment with CPPU compared with those in the bulblet-inducing treatments; and *LohSuSy4* expression showed a nearly opposite trend in the key transition stage (0–15 DAT) within the two different treatments (Figure 7). A similar scenario was observed in *Vicia faba*, where CWINs play regulatory roles in early seed development and SuSy appears to be intimately involved in late seed development (Weber et al., 2005). In potato, SuSy activity markedly increases from the stolon (a type of shoot) stage to visible tuber stage, whereas acid invertase levels decrease (Appeldoorn et al., 1999). Afterward, the change in sucrose phloem unloading from the apoplastic pathway to the symplastic pathway during tuberization was unraveled (Viola et al., 2001), and this change reflects the previously described biochemical trait changes well. On this basis, we propose that bulblet formation might involve a similar unloading pattern shift that drives a successful shoot-to-bulblet transition.

To further test this possibility, the phloem-mobile tracer CFDA was applied to characterize the sucrose unloading pathway. We observed that symplastic unloading clearly predominates during the filling stage in the bulblets (Figure 8), which is consistent with a study in starchy tuberous cassava (Mehdi et al., 2019). Recently, the interaction between a tuberization-specific flowering locus T homolog, StAP6A, and StSWEET11 was revealed to be responsible for regulating the switch in unloading (Abelenda et al., 2019). Our RNA-Seq data regarding the differential expression of unigenes in various treatments annotated to *AtSWEET11* and *AtSWEET12* also suggest the possibility in the abovementioned hypothesis (Supplementary Figure 6). In fact, SuSy contributes more to the synthesis and storage of starch, while CWIN produces more sugars, such as glucose, for cell division in the early developmental stages, probably via the sugar signaling pathway instead of via the provision of carbon (Weber et al., 2005; Baroja-Fernandez et al., 2009; Sonnewald and Fernie, 2018; Liu et al., 2019; Liao et al., 2020). These findings are in agreement with our observations of the sugar and starch contents shown in Figure 4. In other words, a successful shoot-to-bulblet transition is associated with a switch from energy production metabolism to storage metabolism and a parallel shift from invertase-catalyzed to sucrose synthase-catalyzed sucrose cleavage.

### General Model of the Shoot-to-Bulblet Transition in Lily *in vitro*

**FIGURE 9:**
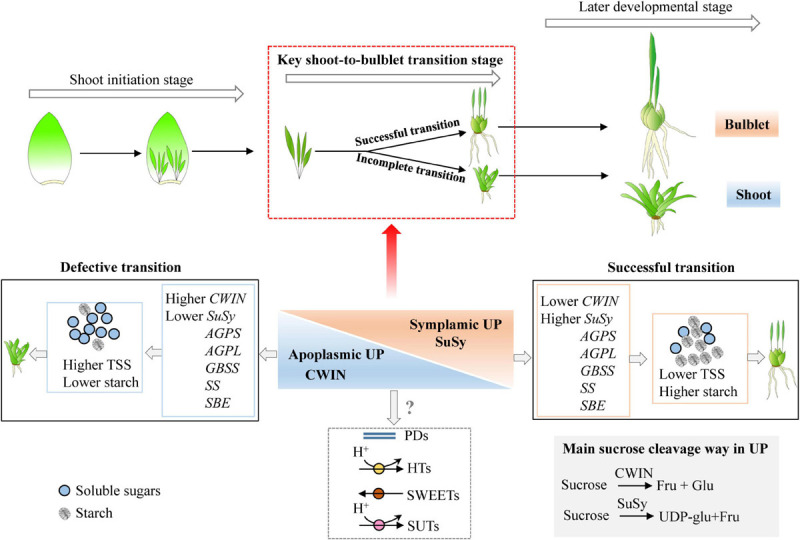
Model of how the shoot-to-bulblet transition is regulated from a starch–sucrose metabolism perspective. The quick starch accumulation driven by high expression levels of starch synthesis-related genes is required during the key transition stage. The starch is therefore the fundamental substance to fill the petiole resulting in bulblet scale. The proper starch synthesis is likely triggered by sucrose cleavage pattern from invertase catalyzed to sucrose synthase catalyzed, which corresponds to a switch from the apoplastic unloading pathway to symplastic one. Nevertheless, the shoot-to-bulblet transition defective treatment is characterized by lower starch content and an incomplete sucrose unloading switch, with higher expression of *LohCWIN* but lower expression of *LohSuSy*. TSS, total soluble sugar; AGPS, adenosine 5′-diphosphate glucose pyrophosphorylase small subunit; AGPL, adenosine 5′-diphosphate glucose pyrophosphorylase large subunit; SS, soluble starch synthase; GBSS, granule-bound starch synthase; SBE, starch branching enzyme; CWIN, cell wall invertase; SuSy, sucrose synthase.

Based on the analysis above, we propose a model of how the different treatments affected the critical shoot-to-bulblet transition stage and resulted in different bulb-formation outcomes (Figure 9). In this model, a switch from apoplastic to symplastic phloem unloading may be involved in the transition process, which is characterized by a reduction in CWIN gene expression and an increase in mRNA levels encoding sucrose synthase, which eventually promote starch synthesis-related gene expression and result in starchy bulblet formation. If these processes do not occur (or are insufficient), the initial shoot will remain as an enlarged shoot. Further studies are needed to obtain a broader spectrum of evidence by using CFDA in real-time imaging to monitor phloem unloading, the ultrastructural basis of the PDs, the PD frequency of the SE/CC complex and its surrounding sink cells (Haupt et al., 2001), and the differential expression of various isoforms of SWEETs (Sosso et al., 2015; Yang et al., 2018), hexose transporters (HTs), and SUTs (Ma et al., 2019; Shen et al., 2019). In addition, invertase and sucrose synthase enzyme activities and sugar (sucrose, glucose, and fructose) levels should be monitored to confirm the activation of the phloem unloading pathway during the key stage (0–15 DAT) and identify the exact signals that confer the ability to transition from shoot to bulblet.

## Data Availability Statement

The datasets generated for this study can be found in online repositories. The names of the repository/repositories and accession number(s) can be found in the article/ Supplementary Material.

## Author Contributions

YX conceived the project. YWu, ZR, and YX designed the experiments. YWu, RM, and MS conducted the experiments. YWu, ZR, CG, SL, DL, and YWe analyzed the data. YWu, ZR, and YX wrote the manuscript with inputs from JW and XW. All authors read and approved the final manuscript.

## Conflict of Interest

The authors declare that the research was conducted in the absence of any commercial or financial relationships that could be construed as a potential conflict of interest.

## Footnotes

1 http://www.ncbi.nlm.nih.gov/gorf/gorf.html

2 https://web.expasy.org/protparam/

3 http://www.cbs.dtu.dk/services/TMHMM/

4 https://swissmodel.expasy.org/

5 http://www.csbio.sjtu.edu.cn/bioinf/Cell-PLoc-2/

6 https://bioinformatics.psb.ugent.be/plaza/

7 http://www.ncbi.nlm.nih.gov
