# Supplementary material for: Change in Sucrose Cleavage Pattern and Rapid Starch Accumulation Govern Lily Shoot-to-Bulblet Transition in vitro
Source: Front Plant Sci. 2021 Jan 14;11:564713. doi: 10.3389/fpls.2020.564713 (PMC7840508; doi:10.3389/fpls.2020.564713)
Supplement: Supplementary file 2 [file Data_Sheet_2.PDF]

Supplementary Material

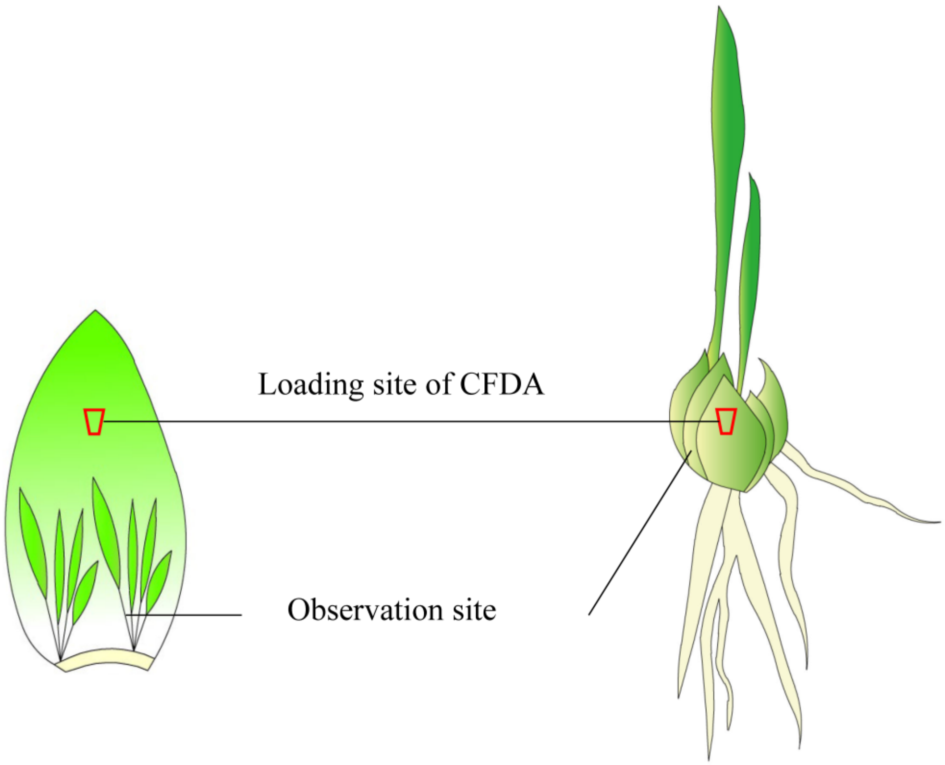

Supplementary Figure 1. Illustrating of 6(5)-carboxyfluorescein diacetate (CFDA) importing.

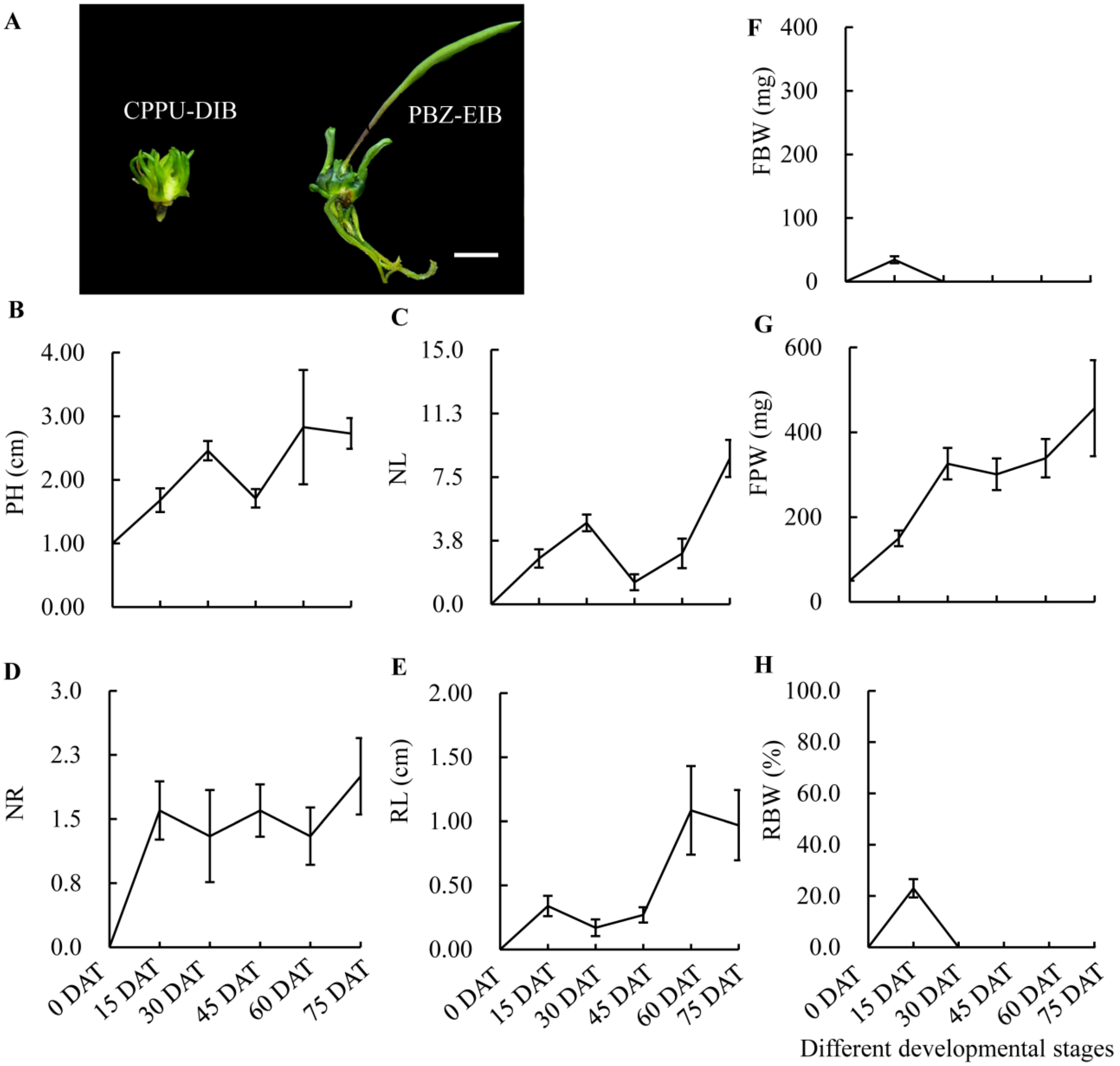

Supplementary Figure 2. Growth status in different treatments in the experimental year 2013. **(A)** Phenotypes recorded at 60 DAT. Bar=1 cm. **(B-H)** The morphological trait changes during developmental stages for CPPU-DIB. Data are represented as the means ± SEM (n=10 biological replicates). PH, Plantlet height; NL, number of leaves; NR, number of roots; RL, root length; FBW, fresh bulblet weight; FPW, fresh plantlet weight; RBW, relative bulblet weight; DAT, days after transplanting; CPPU-DIB, forchlorfenuron-defective in bulbification; PBZ-EIB, paclobutrazol-enlargement in bulbification; CON-NIB, control-normal in bulbification.

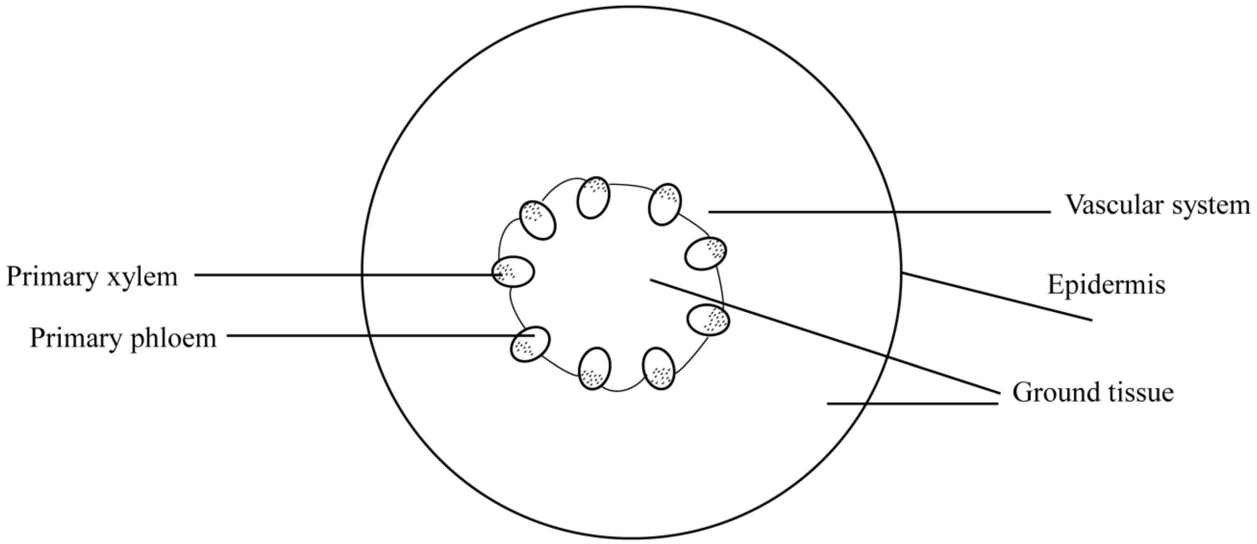

Supplementary Figure 3. Schematic diagram of basal plate lateral section in lily.

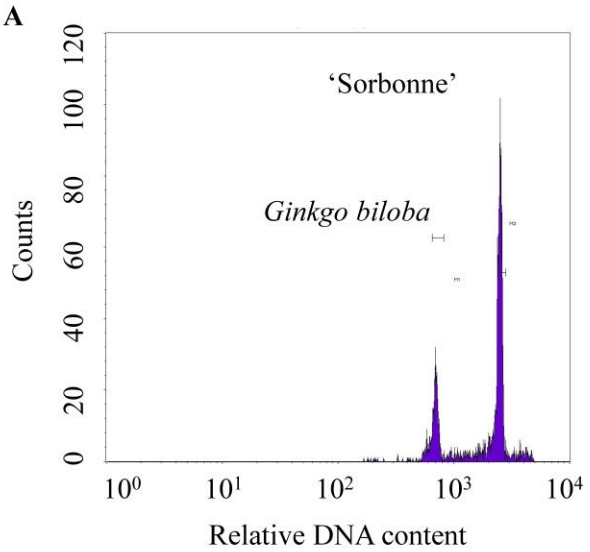

B

Protein	MW(KDa)	pI	TNNCR	TNPCR	GRAVY	II	Predicted locations
LohAGPS1.2b	56.85	6.35	60	57	-0.158	37.05	Chloroplast
LohSSIIId	131.48	5.34	177	136	-0.537	45.25	Chloroplast

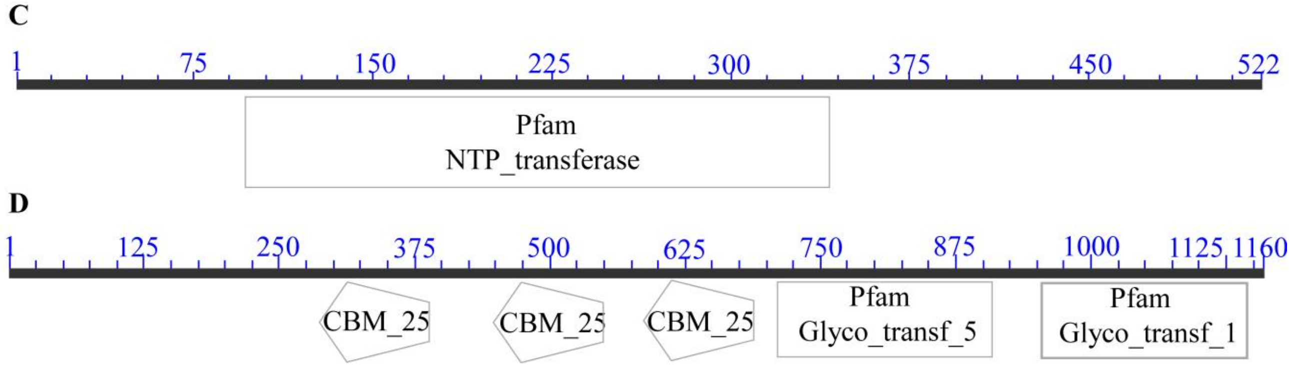

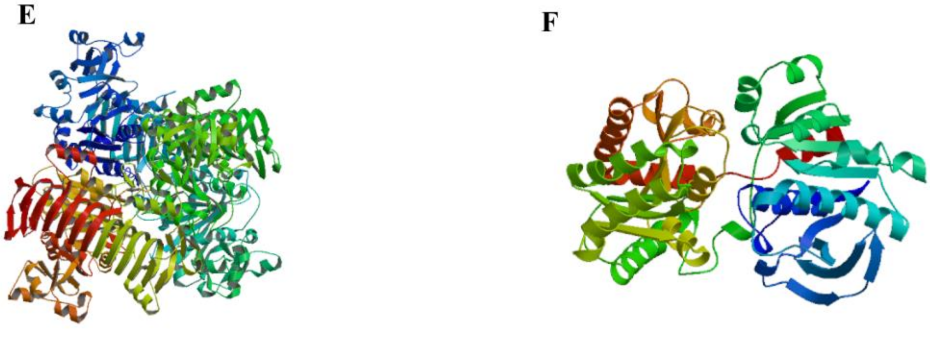

Supplementary Figure 4. Prediction analysis of LohAGPS1.2b and LohSSIIId. **(A)** Histograms of the relative flow-cytometric genome size measurement. The relative fluorescence intensity obtained based on the analysis of propidium iodide-stained nuclei isolated from young leaves of 'Sorbonne'. **(B)** Analysis of protein physical and chemical properties. **(C)** Functional domain analysis. **(D)** Tertiary structure model.

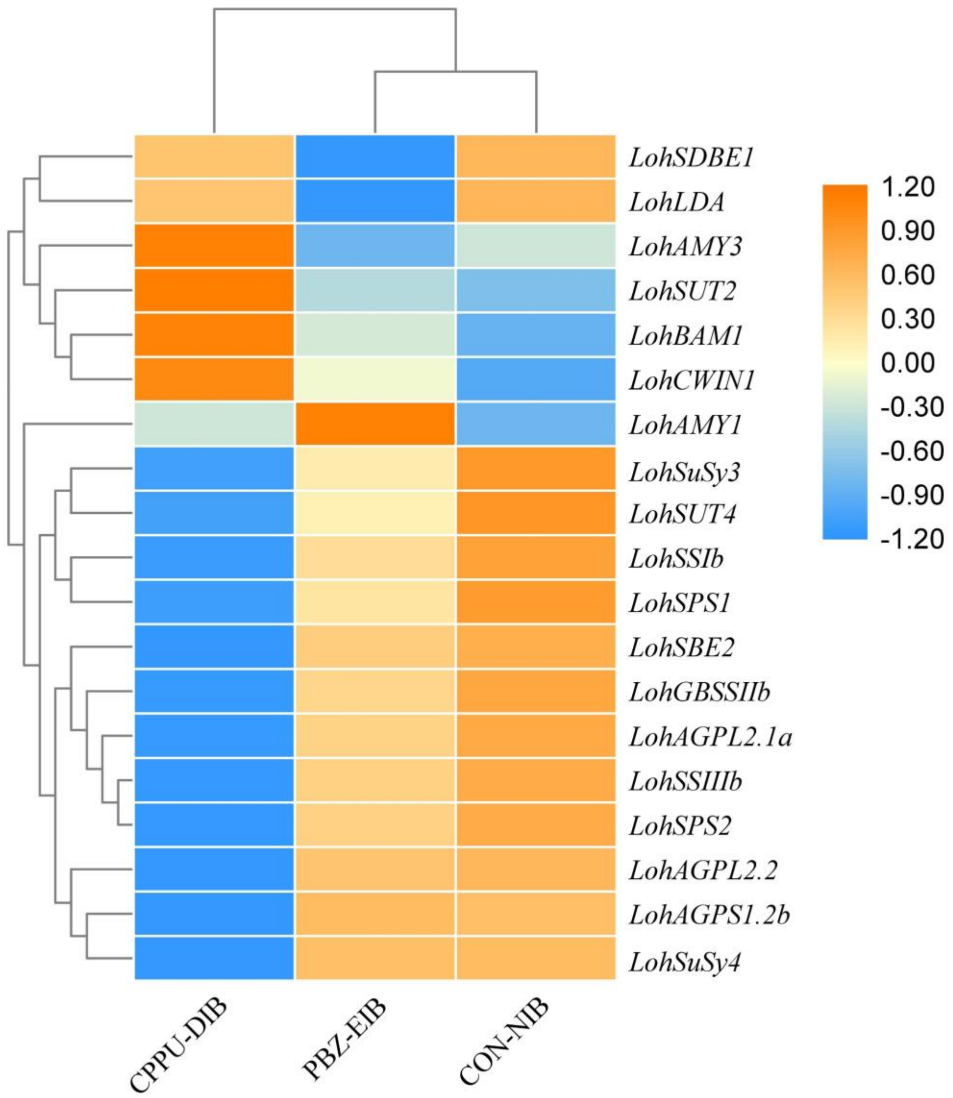

Supplementary Figure 5. Heatmap of the expressed genes according to RNA-seq data in the experimental year 2013 sampled at 60 DAT. The FPKM of the unigenes were presented as fold-change values converted to the log_2 format, and a cluster heat map was generated using TBtools. The expression values were mapped using a color gradient from the low (blue) to high (orange). DAT, days after transplanting; CPPU-DIB, forchlorfenuron-defective in bulbification; PBZ-EIB, paclobutrazol-enlargement in bulbification; CON-NIB, control-normal in bulbification.

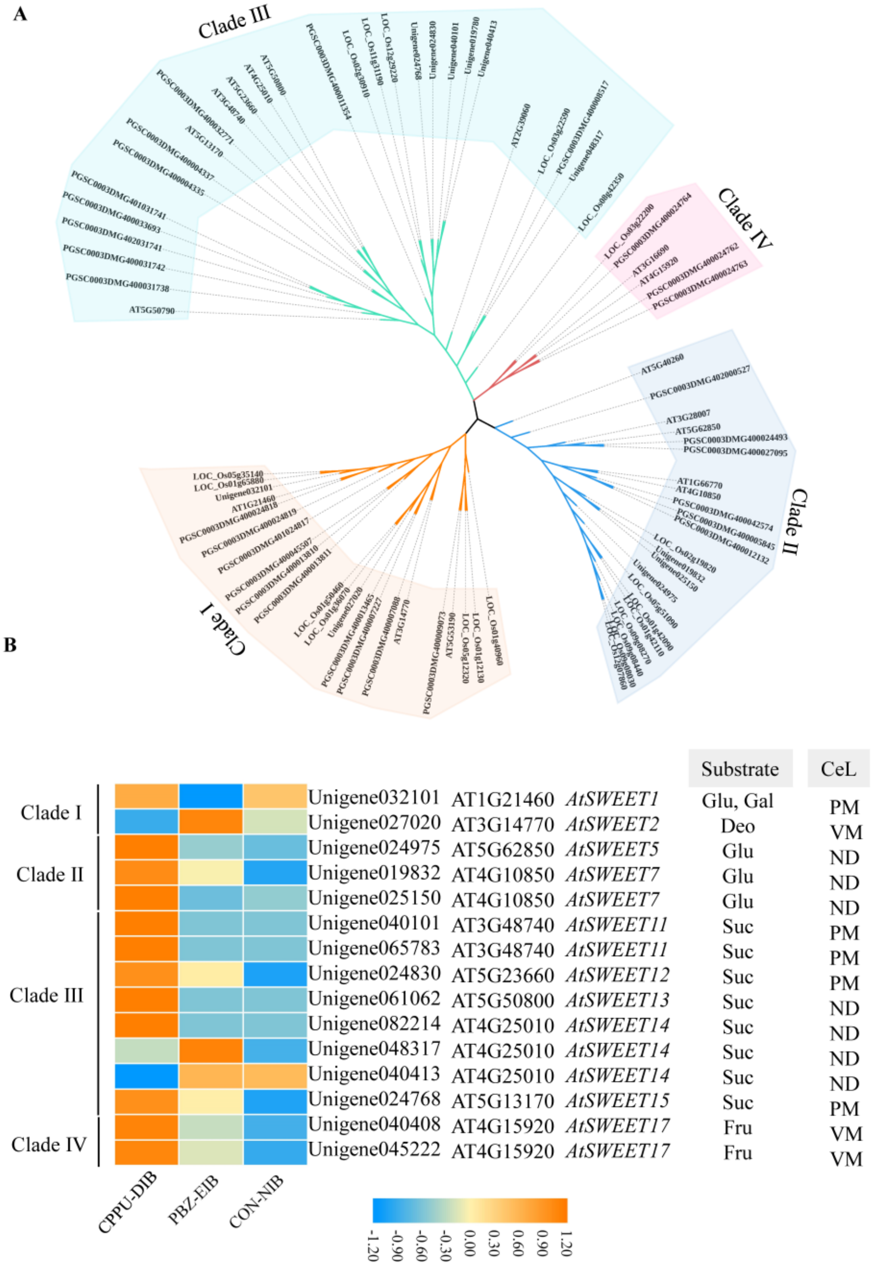

Supplementary Figure 6. The phylogenetic analysis and gene expression heatmap of SWEETs according to RNA-seq data in the experimental year 2013 sampled at 60 DAT. (A) Phylogenetic tree of SWEETS in *Lilium* Oriental hybrids, *Arabidopsis thaliana*, *Solanum tuberosum*, *Oryza sativa* ssp. *japonica* by FastTree software. (B) Heatmap of the expressed SWEETs genes. The FPKM of the unigenes were presented as fold-change values converted to the $\log_2$ format and was generated using TBtools. The expression values were mapped using a color gradient from the low (blue) to high (orange). The corresponding *Arabidopsis* numbers and gene names are listed as well as their substrate and cellular localization (CeL). DAT, days after transplanting; CPPU-DIB, forchlorfenuron-defective in bulbification; PBZ-EIB, paclobutrazol-enlargement in bulbification; CON-NIB, control-normal in bulbification.
